# Comprehensive mPoint: A Method for 3D Point Cloud Generation of Human Bodies Utilizing FMCW MIMO mm-Wave Radar

**DOI:** 10.3390/s21196455

**Published:** 2021-09-27

**Authors:** Guangcheng Zhang, Xiaoyi Geng, Yueh-Jaw Lin

**Affiliations:** 1 School of Mechanical Engineering, University of Shanghai for Science and Technology, Shanghai 200093, China; gxyusst@163.com; 2 College of Engineering and Engineering Technology, Northern Illinois University, DeKalb, IL 60115, USA; ylin1@niu.edu

**Keywords:** mm-wave radar, data processing, human detection, 3D point cloud, comprehensive mPoint

## Abstract

In this paper, comprehensive mPoint, a method for generating 3D (range, azimuth, and elevation) point cloud of human targets using a Frequency-Modulated Continuous Wave (FMCW) signal and Multi-Input Multi-Output (MIMO) millimeter wave radar is proposed. Distinct from the TI-mPoint method proposed by TI technology, a comprehensive mPoint method considering both the static and dynamic characteristics of radar reflected signals is utilized to generate a high precision point cloud, resulting in more comprehensive information of the target being detected. The radar possessing 60–64 GHz FMCW signal with two sets of different dimensional antennas is utilized in order to experimentally verify the results of the methodology. By using the proposed process, the point cloud data of human targets can be obtained based on six different postures of the underlying human body. The human posture cube and point cloud accuracy rates are defined in the paper in order to quantitively and qualitatively evaluate the quality of the generated point cloud. Benefitting from the proposed comprehensive mPoint, evidence shows that the point number and the accuracy rate of the generated point cloud compared with those from the popular TI-mPoint can be largely increased by 86% and 42%, respectively. In addition, the noise level of multipath reflection can be effectively reduced. Moreover, the length of the algorithm running time is only 1.6% longer than that of the previous method as a slight tradeoff.

## 1. Introduction

Human target detection systems are widely employed in various areas for specific purposes such as safety, healthy and energy conservation. For the application in smart vehicles, human detection technology can aid in avoiding collisions around the vehicle and provide child-left-behind warning to drivers, which enhances safety and security. For applications in smart homes, it is helpful to offer comfortable living environments and to improve the quality of living by controlling temperature, humidity, noise, light and air quality by detecting and tracking people in a room [1]. In addition, the incomplete list also includes military applications, service robots and search and rescue after severe disasters [2,3].

In recent years, numerous sensors have been applied to achieve contactless detection, tracking and classification of human targets [4,5]. Conventional sensors are utilized to detect occupancy in a specific area, such as passive infrared (PIR) sensor, CO_2_ sensor and ultrasonic sensor [6,7,8,9]. However, these sensors have the limitations such as sensitivity to temperature, slow response, etc. The vision-based system takes the advantage of ultra-high resolutions and can be implemented easily. However, its light sensitivity and privacy violation also limit the promotion of the system in some other applications [10,11,12].

Compared to the above sensors, the millimeter-wave radar sensor has the advantages of long-range capabilities, low cost and the ability to work in non-line-of-sight situations, such as through building walls and clothes [4,13,14]. It was proved that the mm-wave radar is robust against non-line-of-sight interference including foam, plastic, etc., with less than 1% change in point-cloud density [15].Moreover, it is a form of non-intrusive technology and does not cause privacy issues, which is particularly valuable [16]. Thus, millimeter-wave radar sensors currently attract increasing attention from both academia and the industry [17,18,19,20,21].

In order to apply the mm-wave radar in practical applications, the relevant data features must firstly be extracted from the reflected signal [22,23,24,25,26]. Hence, data processing methods for extracting more detailed features from the reflected signal, such as the features of the distance [27], velocity [28,29], Radar Cross Section (RCS) value [30] and angle [31], are widely investigated. Radar point cloud data not only contains almost all the aforementioned features but also can directly indicate the spatial locations of the targets, and they are receiving more attention. However, most of these research investigations are focused only on feature extraction and recognition after obtaining point clouds without paying much attention to the generation of the point cloud [15,32,33,34,35,36]. This causes inaccurate results because it is well known that the quality of the generated point cloud has a significant effect on the accuracy and effectiveness of the subsequent data process.

As it is well known, high angle resolutions that can generate dense point clouds are closely related to the antenna array with a large aperture in both azimuth and elevation. However, the cost of the hardware increases along with the number of antenna elements. One possible solution to reduce costs without sacrificing the angle resolution is by utilizing MIMO radar [37,38,39]. For example, the MIMO radar with two-dimensional sparse arrays and hundreds of virtual elements can enable high-fidelity four-dimensional sensing (range, Doppler, azimuth and elevation) [40].

As for the solutions of data processing from the FMCW-MIMO radar raw data to the target point cloud, the 3D-Fast Fourier Transform (FFT) algorithm still occupies the mainstream position [41]. The algorithm was clearly illustrated in related investigations, for example, the FFT is performed firstly on the data of three different dimensions (radar signal in time domain, chirp and antenna) one after another; on the other hand, velocity and angle of the target are determined as a result [42,43]. Common peak detection methods such as threshold-based methods and Constant False Alarm Rate (CFAR) are applied between FFT signal processing to finally generate the spatial point cloud. However, the point cloud generated is too sparse and cannot distinguish the difference between the background environment and human targets.

**Figure 1:**
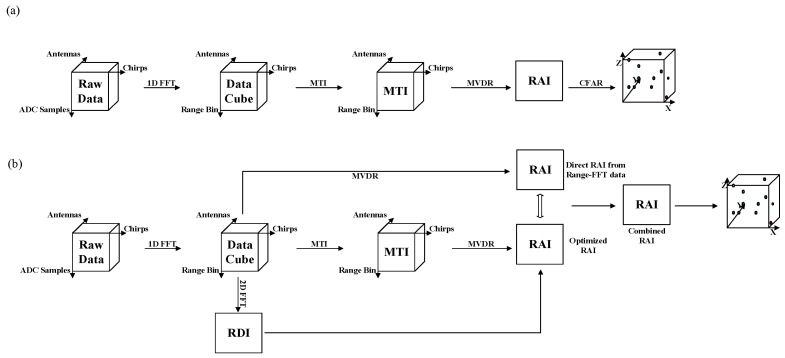
The radar signal processing flows: (**a**) TI-mPoint and (**b**) proposed comprehensive mPoint.

Recently, the method proposed by Texas Instrument Technology (TI-mPoint) is widely used in human detection, and its strategy is shown in Figure 1a. The order of the data process could be simplified as the Range-FFT, moving target indication (MTI) method [44] and Minimum Variance Distortionless Response algorithm (MVDR) [45], and it can capture the Range Angle Image (RAI) of the target and detect the peak points by the Constant False Alarm Rate (CFAR) [46,47]. At the end, the human target 3D point cloud is obtained, which is denser and more accurate than the aforementioned method by introducing Digital Beamforming (DBF) technology (get more information on TI-mPoint, please refer to https://training.ti.com. Accessed on 25 August 2021). Since the MTI process is added in the TI-mPoint, the point cloud of the human targets could be separated from the background, which extended the application of the mm-wave radar [15,35]. However, due to the multipath reflections caused by other objects in the background during the MTI process, noise points badly appear in the point cloud. Moreover, since the MTI results show the intensity of the motion of the target and not the true intensity of human body reflection, the point cloud generated by TI-mPoint cannot reflect true spatial information of the human body that is accurately detected.

In this paper, a novel method (comprehensive mPoint) for generating 3D point cloud of human targets, considering both the static and dynamic characteristics of radar reflected signals, is proposed and illustrated in Figure 1b. Firstly, by introducing the Range Doppler Image (RDI), the multipath refection noise in the RAI from MTI result is reduced, and the optimized RAI is obtained; then, direct RAI from Range FFT data is obtained with the help of MVDR, and the more detailed feature of the direct RAI is located and extracted by combining the optimized RAI, namely combined RAI. Finally, 3D point cloud can be produced as a result of the combined RAIs. Subsequently, the results of the methodology and the length of the algorithm running time were verified in six different human postures based on the defined point cloud accuracy rate. Compared with the TI-mPoint, the point numbers and accuracy rate of the point cloud generated by our proposed comprehensive mPoint increased by 86% and 42%, respectively. The comparisons will be explained in the paper.

The innovative contributions are summarized as follows:

1. In this paper, a novel data processing method considering both the static and dynamic characteristics of radar reflected signals is proposed and utilized in order to generate the point cloud of the human bodies.
2. The RDI is introduced to help reduce noise during the radar signal target detection process in order to improve the performance of angle estimation on MTI results. The optimized RAI is obtained.
3. The optimized RAI is utilized to locate the target on the direct RAI from Range-FFT data. More detailed reflection information of detected target is obtained from the combined RAI.
4. Both the azimuth and elevation angle information of the detected target from two combined RAIs are captured based on the corresponding range and SNR value in order to help generate the 3D point cloud of the target.

## 2. Background Theory and Proposed Method

**Figure 2:**
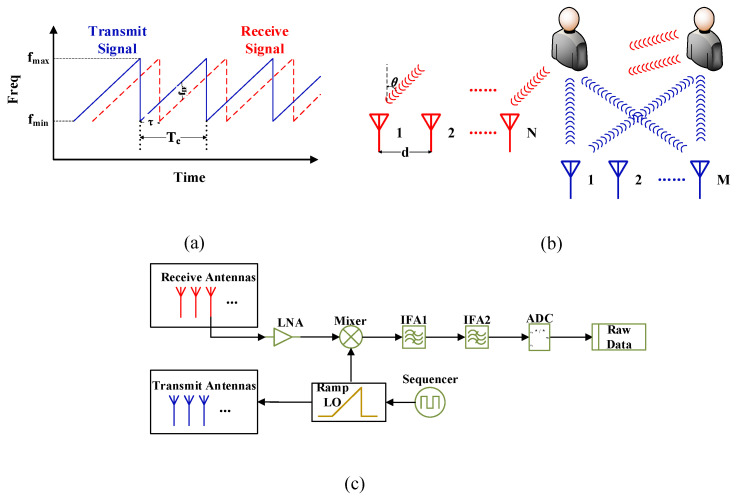
(**a**) The time-frequency diagram of the FMCW signal, (**b**) the MIMO antenna structure and (**c**) the FMCW signal chain of the radar.

Figure 2 shows the transmitted signal of the FMCW-MIMO mm-wave radar utilized in the study. A chirp signal is the FMCW signal in a cycle $T_{c}$. In each chirp signal, the radar uses the Time-Division Multiplexing (TDM) MIMO Angle-Of-Arrival (AOA) estimation algorithm to increase the angular resolution by M transmit antennas and N receive antennas, as shown in Figure 2b.

Mathematically, a transmitted chirp signal and the corresponding received signal is given by the following:

(1) $$s_{T}\left(t\right)={\;A}_{t}\exp\;\left(j\left(2{\pi f}_{\min}\;t\;+\;\pi \frac{B}{T_{c}}t^{2}\right)\right),\;0\;<\;\;t\;<\;{\;T}_{c}$$

(2) $$s_{R}\left(t\right)={\;A}_{r}\exp\;\left(j\left(2{\pi f}_{\min}\left(t-\tau \right)+\pi \frac{B}{T_{c}}{\left(t-\tau \right)}^{2}\right)\right)\;$$

(3) $$s_{\mathrm{IF}}\left(t\right)={\;s}_{T}\left(t\right){*s}_{R}\left(t\right)={\;A}_{t}A_{r}\exp\left(j\left(\frac{4\pi \mathrm{BRt}}{{\mathrm{cT}}_{c}}+\frac{4\pi R}{\lambda }\right)\right)$$

where $A_{t}{\;\mathrm{and}\;A}_{r}$ are the amplitudes of the signals, $f_{\min}$ is the initial sweep frequency, B is the sweep bandwidth and τ =2R/c is the time delay between the transmitted signal and the received signal reflected by the target at the distance R. Two signals are mixed to obtain the Intermediate Frequency (IF) signal, which is related to the range of target.

Although FFT processes data of multiple dimensions, Range FFT data including range information and RDI including velocity information are calculated [33]. The frequency of IF signal is $\begin{array}{c} f_{\mathrm{IF}}=\frac{2\mathrm{BR}}{{\mathrm{cT}}_{c}} \end{array}$, the range resolution is $\Delta R\;=\frac{c}{2B}$, the maximum unambiguous velocity is $v\;=\frac{\lambda }{4T_{c}}$ and the velocity resolution is $\Delta v\;=\frac{\lambda }{2T_{c}f_{n}}$, where λ is the wave length of the radar signal, and $f_{n}$ is the number of chirps. In former studies, RDI was mostly utilized to estimate the velocity of the targets. However, its implementation in noise reduction has received little attention.

To determine the AOA of the target, the MVDR algorithm, which has better resolution than Angle-FFT, is introduced [48]. In this paper, the receive antenna is assumed as a linear antenna array possessing N receive antennas, and the received signal of the first receive antenna is $s_{r}\left(t\right)$; the received signal is given by the following:

(4) $$\begin{array}{c} x\left(t\right)={\;s}_{r}\left(t\right)*a\left(\theta \right) \end{array}$$

where a(θ) is the steering vector $\;{\left[1,\;e^{j\frac{2\pi d\sin\left(\theta \right)}{\lambda }},{\;e}^{j\left(\left(N-1\right)\frac{2\pi d\sin\left(\theta \right)}{\lambda }\right)}\right]}^{T}$. By using the maximum likelihood method, the power distribution at different angles is calculated as follows:

(5) $$P\left(\theta \right)=\frac{1}{a{\left(\theta \right)}^{H}R^{-1}a\left(\theta \right)}$$

where $R\;={\;x}_{t}{*x}_{t}^{H}$, the angle information (RAI) of the targets is obtained.

In order to detect more comprehensive information of the target by using radar data, the data process method proposed here considers more data features, as shown in Figure 1b.

Firstly, the captured raw radar cube data are regarded as three-dimensional (radar signal in time domain, chirp and antenna) cube data. After Range FFT data and RDI are calculated, the MTI process is applied to remove static information in the received signal; dynamic information (DI) is calculated to highlight movement information existing in the target in which $\mathrm{FFT}\left({\mathrm{chirp}}_{i}\right)$ is the FFT result of the ith chirp signal.

(6) $$\;\mathrm{DI}\;=\overset{f_{n}}{\underset{i=1}{\sum }}\left(\mathrm{FFT}\left({\mathrm{chirp}}_{i}\right)-\overset{f_{n}}{\underset{i=1}{\sum }}\frac{\mathrm{FFT}\left({\mathrm{chirp}}_{i}\right)}{f_{n}}\right)$$

Next, the MVDR algorithm is applied to obtain the direct RAI from Range-FFT data and the RAI from MTI result, respectively. Afterward, the RDI and two different RAIs are combined to obtain more detailed information and the point cloud of the target as stated below:

- Detect the peak value of velocity in RDI and determine the distance R of the human target.
- Find the moving target information at R in the RAI from MTI result, which is represented by area A. Area A refers to an area in the Range–Angle plane where the moving target is located, such as the target area in the RAI.
- Corresponding to the target information in the RAI from MTI result, the data at the corresponding position (area A) in the direct RAI from Range-FFT data is used as the target data, which is $A^{*}=\left\{P_{\left(\mathrm{range},\;\mathrm{angel},\;\mathrm{SNR}\right)}\right\}$. The set $A^{*}$ is a set of target points P on the angle plane, and it includes the distance, angle and signal-to-noise ratio values.
- After obtaining the target data $A_{1}^{*}=\left\{P_{\left(\mathrm{range},\;\mathrm{azimuth}\;\mathrm{angle},\;\mathrm{SNR}\right)}\right\}$ on the azimuth plane and $A_{2}^{*}=\left\{P_{\left(\mathrm{range},\;\mathrm{elevation}\;\mathrm{angle},\;\mathrm{SNR}\right)}\right\}$ on the elevation plane, the point cloud of the target is finally produced based on the corresponding range and SNR value on different angle planes. The formula is given by the following:
  (7) $$A_{1}^{*}⊕A_{2}^{*}\to \left\{P_{\left(\mathrm{range},\;\mathrm{azimuth}\;\mathrm{angle},\;\mathrm{elevation}\;\mathrm{angle},\;\;\mathrm{SNR}\right)}\right\}$$
  where ⊕ represents the combination of target data in two different angle planes. The above algorithm process can be represented in the following pseudocode (Algorithm 1).

**Algorithm 1:** **Point Cloud Generation****FUNCTION** Combined RAI Generation (data = Radar Raw Data):   Range-FFT data = **1D FFT** (data), RDI = **2D FFT** (Range-FFT data)   MTI result = **MTI** (Range-FFT data), RAI from MTI result = **MVDR** (MTI result)   Direct RAI = **MVDR** (Range-FFT data)   **For** d (range bin, velocity) in RDI:     **If** d> SNR:       range bin(d) ∈ speed change area   **If** d **in** no speed change area:     Optimized RAI = RAI from MTI result(d = 0)   **For** d (range bin, velocity) in optimized RAI:     If d > SNR:       Combined RAI = Direct RAI (d) **Return** combined RAI combined RAI (azimuth), combined RAI (elevation) = **Combined RAI Generation** (data) **For** d1 (range bin, azimuth angle bin) in combined RAI (azimuth):  **If** d1 > SNR:    **For** d2(range bin, elevation angle bin) **in** combined RAI (elevation):      **If** range bin(d2) = range bin(d1) **And** d2 = d1:        Point cloud **add** p (range, azimuth angle, elevation angle, SNR) **Return** Point cloud **END**

The comprehensive mPoint proposed here considers more data features of the following: The velocity information of the target in RDI is considered creatively for noise reduction in order to obtain the optimized RAI; not only the position of target is located from the optimized RAI but also the directly reflected information in the RAI from Range-FFT data is regarded as the target data; the target data in the combined RAIs of two different angle planes is correlated in order to produce point clouds based on the fact that the target’s reflection points in azimuth and elevation planes have the same range and SNR values.

## 3. Experimental Implementation, Result and Discussion

### 3.1. Radar Sensor and Testbed Setup

**Figure 3:**
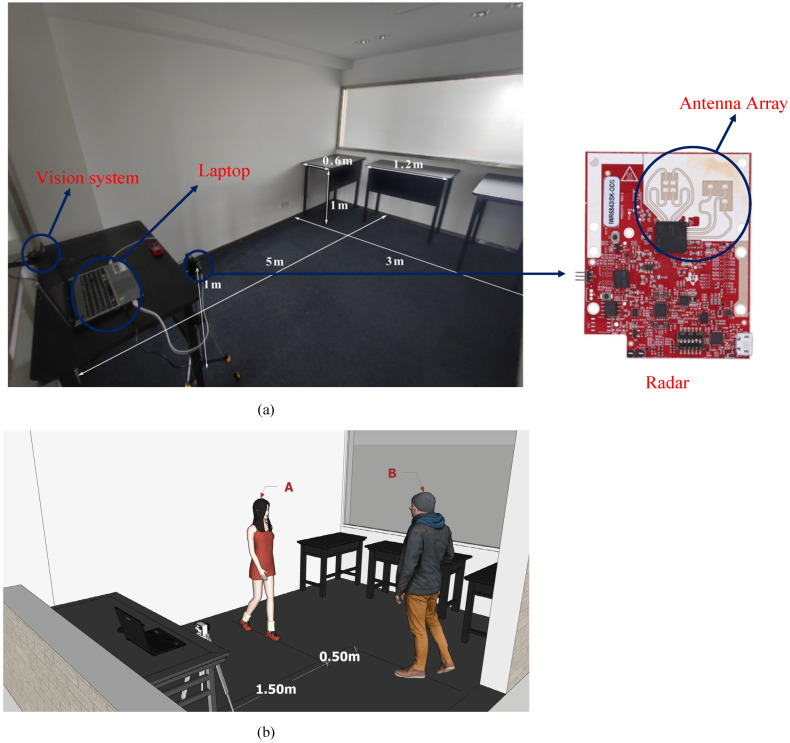
Experimental setup: (**a**) the office room where the experiment conducted and (**b**) the room with various objects.

The system is developed on a commercial millimeter wave radar sensor IWR6843ISK-ODS (Texas Instruments) using three transmit antennas in L-shaped configuration and four receive antennas in rectangle-shaped configuration. The two-dimensional antenna array shown in Figure 3 has 120 degrees range of view at both the azimuth plane and elevation plane. The detailed configuration of the radar parameters is shown in Table 1. The specific test environment is set up in an office room, as shown in the Figure 3a, where the radar board is located 1 m away from the ground. The sensor data are transferred to the computer through the DAQ (FPGA board DCA1000). All data processing of the system is implemented in the computer, which receives the raw data collected by the radar and then generates a 3D point cloud.

### 3.2. Point Cloud Generation with Two Moving Targets and Discussion

To better explain the comprehensive mPoint, the experimental environmental is set up (Figure 3). The test environment is a 3 × 5 m room. The radar is located in the left center of the room and 1 m away from the ground. There are three iron tables evenly distributed on the right side of the room, and the size of the table is 1.2 × 0.6 × 1 m. Testers A and B are pacing around 1.5 m and 2.0 m in front of the radar, where A is 1.64 m tall and weighs 52 Kg and B is 1.75 m tall and weighs 75 Kg.

**Figure 4:**
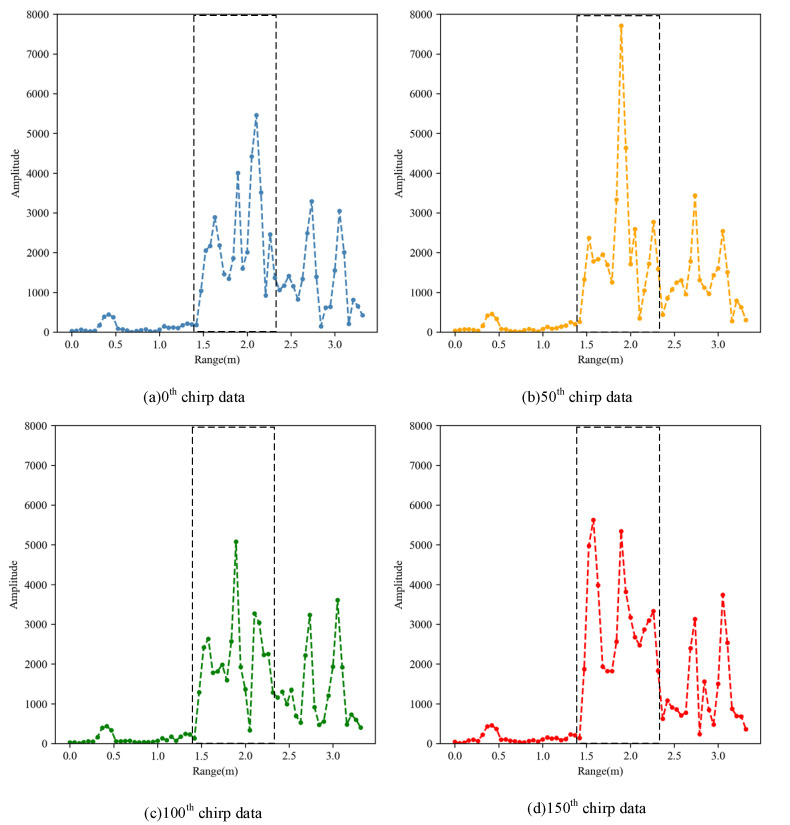
The results of Range-FFT for four selected chirps signals. (**a**) 0th chirp data, (**b**) 50th chirp data, (**c**) 100th chirp data and (**d**) 150th chirp data.

**Figure 5:**
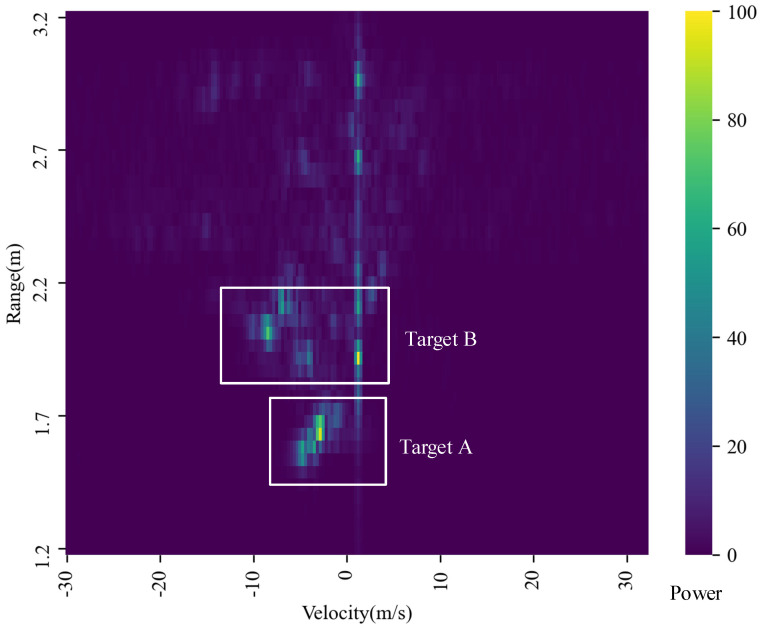
The result of Doppler-FFT (RDI).

The results of the Range-FFT are shown in Figure 4; there are obviously several peaks in the Range-FFT data that correspond to the different distance of the targets in four selected chirp signals. In the experiment, two testers are 1.5 m and 2 m away from radar respectively, but due to the reflection characteristics of the human body, the two peaks appear around 1.5 ± 0.3 m and 2.0 ± 0.3 m, respectively. In addition to these peaks, there are also other FFT peaks caused by other indoor objects such as tables, walls, etc., that will be suppressed after the MTI process. Then, Doppler-FFT result RDI is shown in Figure 5. The bright spots illustrate the reflection of targets with the abscissa corresponding to the velocity of the targets. There are obviously some areas brighter than the surrounding area between 1 m and 2.2 m corresponding to the higher power data, which was clearly caused by the motion of human body. In this paper, peak detection based on threshold is used in both RDI and RAI, where the data value is mapped to the interval of 0–100, and 50 is selected as the high-power value threshold after multiple tests. Thus, the range of human targets can be determined.

**Figure 6:**
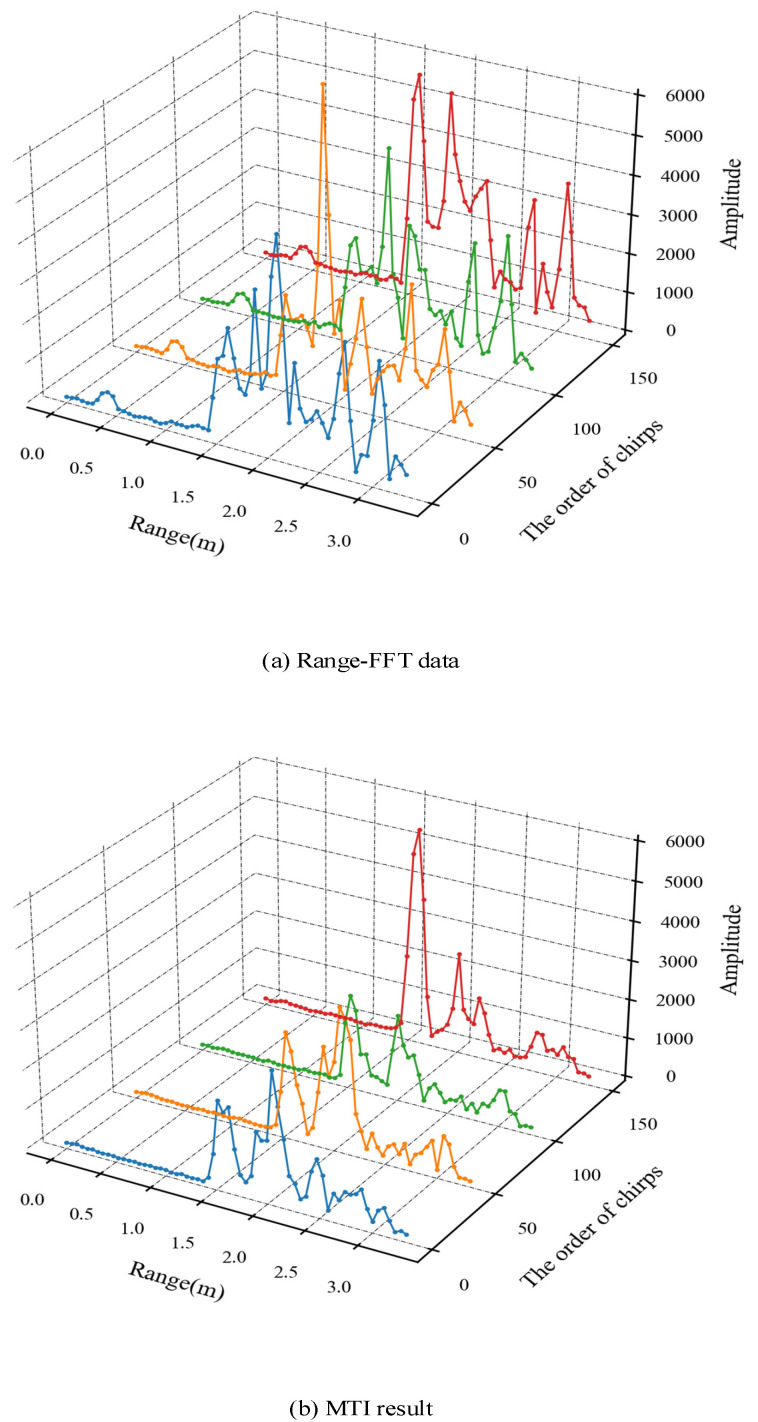
The results of (**a**) Range-FFT and (**b**) MTI process.

The result of the MTI process is shown in Figure 6 in which part (a) shows the Range-FFT result and (b) shows the MTI result; this clearly shows that the peaks values caused by other static backgrounds in the room (such as tables, walls, etc.) are effectively suppressed compared with Figure 6a at 0–1.5 m and 2.5–3 m. It is worth noting that the vertical axis amplitude value in (a) and (b) reflects the intensity of the human body reflection and the intensity of the human movement, respectively.

**Figure 7:**
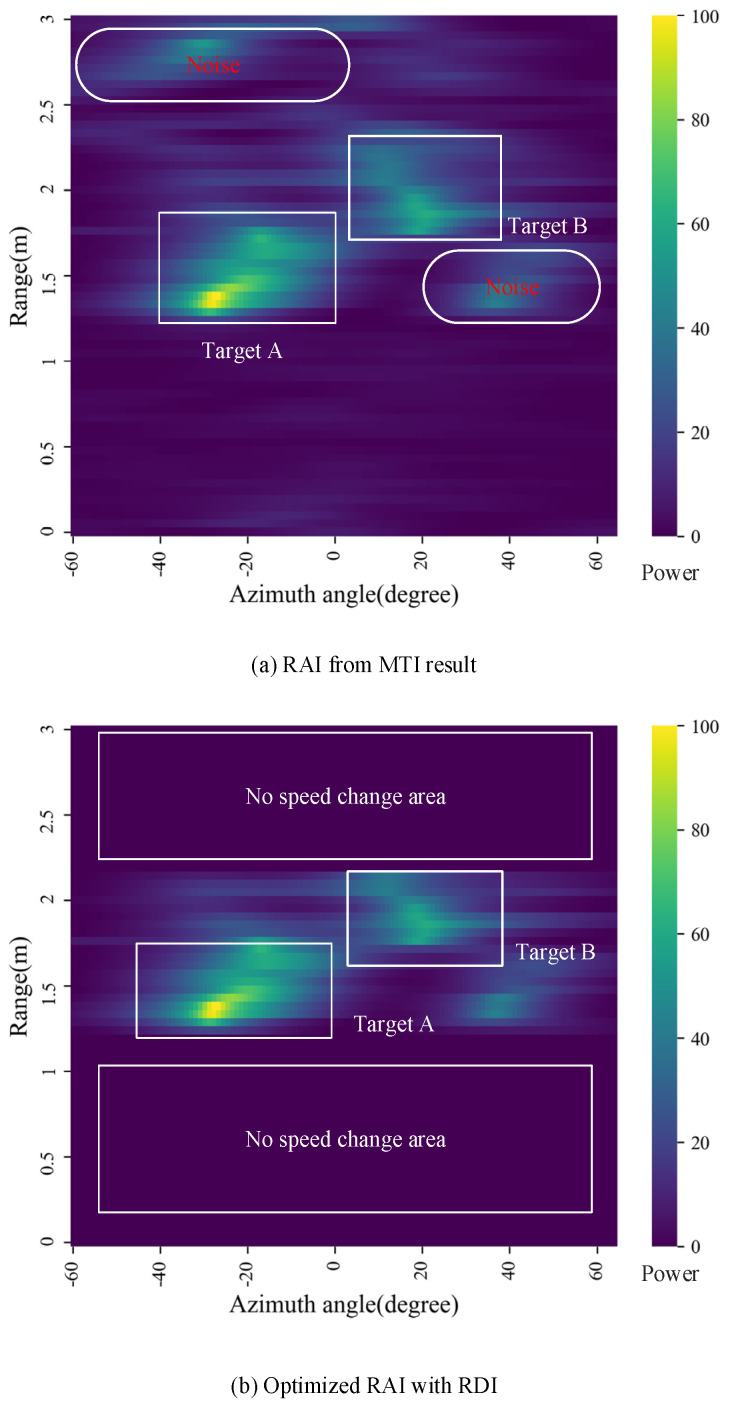
Angle images: (**a**) RAI from MTI result and (**b**) optimized RAI with RDI.

**Figure 8:**
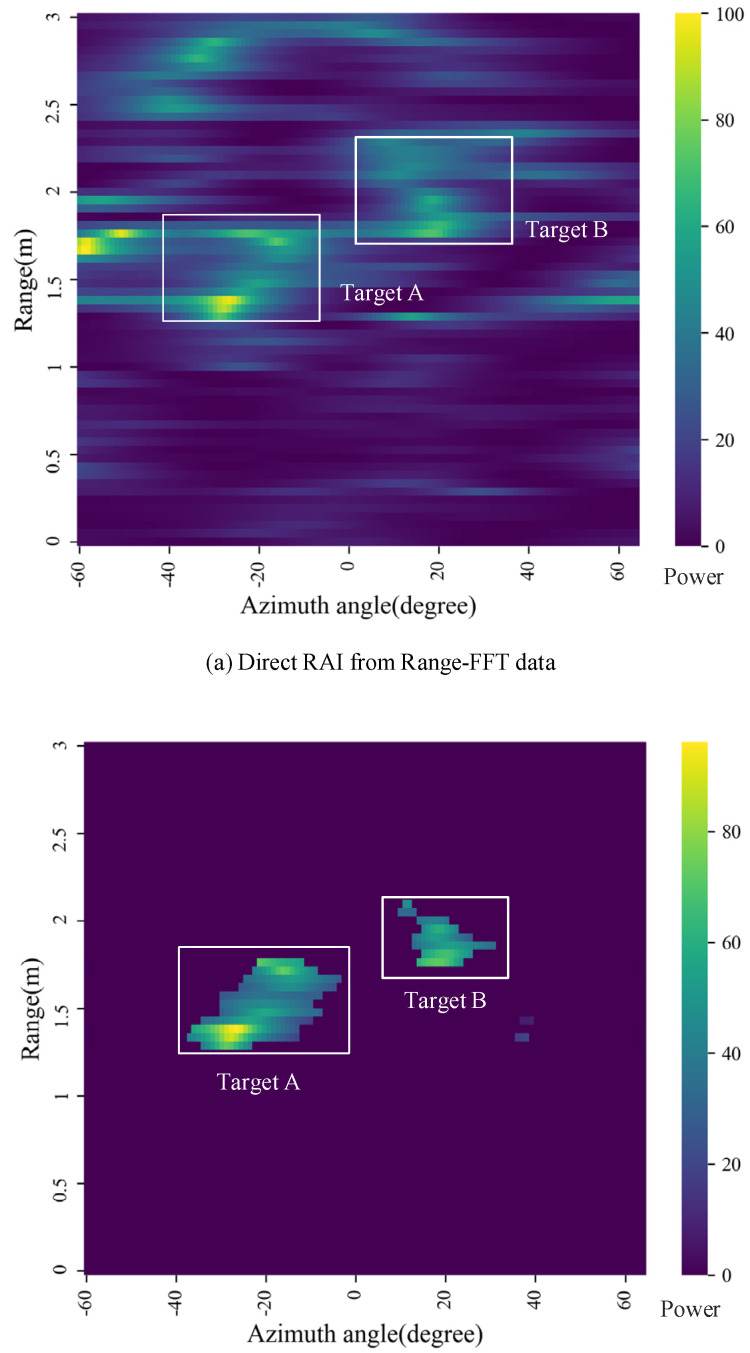
Angle images: (**a**) Direct RAI from Range-FFT data and (**b**) combined RAI.

**Figure 9:**
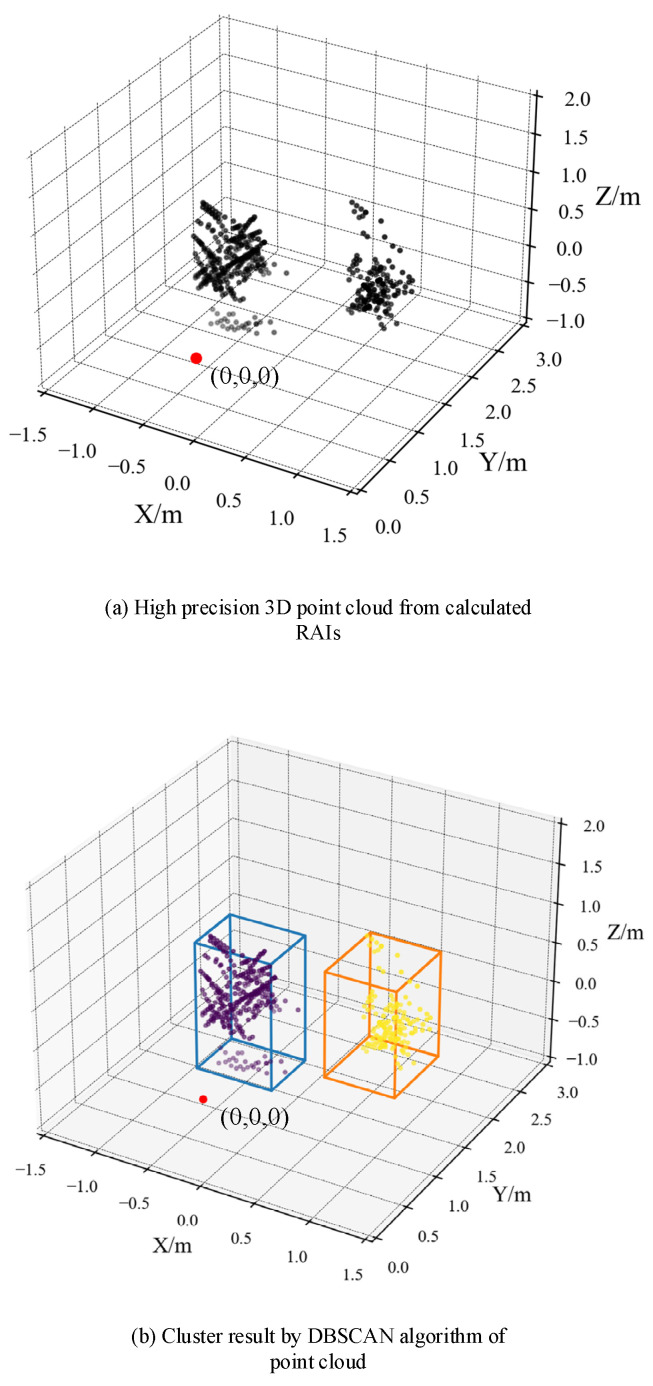
(**a**) Generated point cloud and (**b**) cluster result.

According to the comprehensive mPoint method introduced in Figure 1b, the results in Figure 7, Figure 8 and Figure 9 can be obtained, respectively. After the MTI and MVDR process, the dynamic characteristics of the reflected signals and the optimized RAI were obtained and shown in Figure 7. Direct RAIs including static characteristics of the reflected signals were obtained from Range FFT data and shown in Figure 8a. The combined RAI shown in Figure 8b is the integrated result of optimized RAI and direct RAI. Finally, the 3D point clouds were generated from the combined RAI of two different angle planes, and the clustering results are shown in Figure 9a,b.

The RAIs from MTI result and the optimized RAI are shown in Figure 7. Two human targets are marked out in both RAI (Figure 7a) and RAI (Figure 7b) through the MTI process, and most backgrounds noises were reduced effectively, but the noise due to the multipath reflection still remains in the Figure 7a. With the help of RDI, this kind of noise in areas with no speed change can further be reduced, as shown in Figure 7b. In addition, some noise is also reduced in peak detection process, for example, the noise signal under target B in Figure 7a is suppressed in Figure 7b since the power value is lower than the SNR threshold.

In Figure 8a shows the direct RAI from Range FFT data: The human targets are aliased with other static backgrounds. In Figure 8b, the combined RAI and the target data on the azimuth plane are finally determined by combining two different RAIs together. The contours of the two human targets are clear enough to show the features of the targets. Moreover, it is easily to understand that the target data on elevation plane is similar with that shown in Figure 8b, but it occupies more angle bins in the elevation angle direction because the azimuth plane represents the width and the elevation plane represents the height of the human target. The RAI representing the intensity of movement of the human body is shown in Figure 7a, the RAI representing the intensity of the human body reflection is shown in Figure 8a and the combined RAI that considers both the static and dynamic characteristics of the human body is shown in Figure 8b. Thus, the combined RAI that includes accurate location and reflection intensity is the more comprehensive human feature.

Then, the point cloud was produced from two RAIs of different angle plane and is shown in Figure 9a, where the red dot (0, 0, 0) is the position of the radar. The generated point clouds of target A and target B are accurately aligned with the human targets’ position from the vision system.

In addition, in this paper, in order to detect the targets in the experiment, the density-based cluster algorithm DBSCAN is used to detect distinct human targets and to separate the human targets from noises that may still remain. The DBSCAN algorithm is attractive because it does not need the number of clusters to be a priori specified, and it can mark outliers as the noise automatically. There are two parameters in the algorithm: Epsilon, which indicates the maximum distance of two points in the same cluster, and MinPts, which indicates the minimum point number to initialize a new cluster. In this study, the proper parameters 0.5 as Epsilon and 20 as MinPts are chosen for the algorithm. The cluster result is shown in Figure 9b, the point cloud is divided into two clusters that represent the two targets and there are no outliers in the result.

The 3D point cloud of the human targets is finally produced based on the proposed comprehensive mPoint, which is consistent with the test environment. Moreover, the cluster algorithm DBSCAN can be easily applied on the point cloud to detect distinct human targets.

### 3.3. Characteristics of the Generated Point Cloud and Discussion

**Figure 10:**
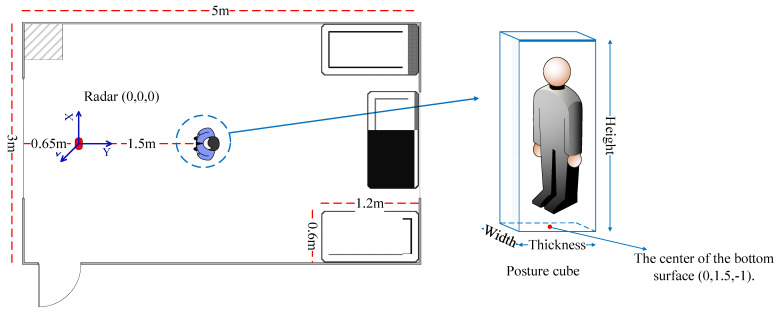
Experimental setup for human target posture and the posture cube.

In order to discuss the performance of the comprehensive mPoint, six sets of radar raw data of target B including Standing, Sitting, Lying, Horse stance, Lunge and Hands up postures are collected in the environment, as shown in Figure 10. The background environment is same as that shown in Figure 3, and the person involved in the test stands 1.5 m directly in front of the radar.

The concept of the accuracy rate of the human target point cloud is proposed and described to estimate the accuracy of the generated point cloud in this paper: $A_{c}=\frac{P_{\mathrm{in}\;\mathrm{cube}}}{P_{\mathrm{sum}}}$ where $P_{\mathrm{in}\;\mathrm{cube}}$ is the point number of point cloud within the posture cube, and $P_{\mathrm{sum}}$ is the total number of the generated point cloud. In terms of posture cube, corresponding to each test posture, the spatial position of the human body is different and marked by a cube with height, width and thickness, respectively, and the center of the bottom surface of the cube is located in at the coordinate (0, 1.5, −1) (shown in Figure 10). The parameters of each body posture cube are given and shown in Table 2. It is common to understand that the higher the value of $A_{c}$, the higher the accuracy of the generated point cloud. In addition, the point cloud accuracy rate mentioned in the following text refers to the parameter Ac that is defined here.

**Figure 11:**
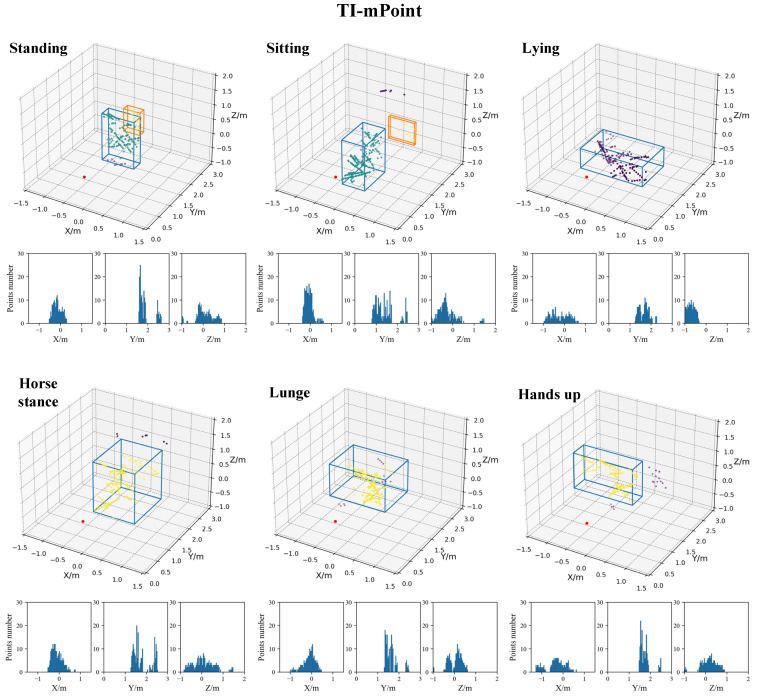
The results of point cloud based on TI-mPoint with 3D point cloud and three-dimensional distribution histogram.

**Figure 12:**
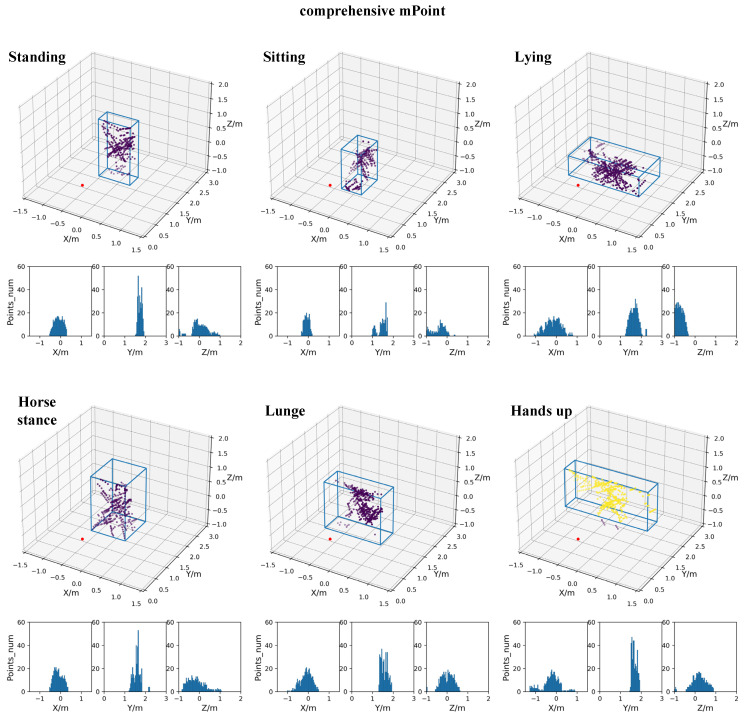
The results of point cloud based on the proposed comprehensive mPoint with 3D point cloud and three-dimensional distribution histogram.

In order to compare the characteristics of two data processing methods, the TI-mPoint and the comprehensive mPoint are both utilized to generate target point clouds. The point cloud results based on TI-mPoint, which include 3D point cloud and its three-dimensional distribution histogram, are shown in Figure 11. While the results of comprehensive mPoint are shown in Figure 12. Figure 11 and Figure 12 demonstrate the effectiveness of the comprehensive mPoint. Compared with TI-mpoint, the results of comprehensive mPoint in this paper have a denser point cloud with fewer noise points. For example, there are two clusters in standing and sitting postures in Figure 11, while Figure 12 has only one cluster.

The clustering results of Standing and Lying postures are vertical and horizontal cubes, respectively, which is same as the actual situation; hence, the generated point clouds by both methods are both consistent with the true posture, but it is intuitively shown that comprehensive mPoint results have denser points and less noise points than the TI-mPoint result. For example, in the TI-mPoint result, the point cloud is sparse and even includes multipath reflection noise, which causes ghost images, but the result of the comprehensive m-Point method shows that the generated point cloud is denser, and there is no ghost image.

**Figure 13:**
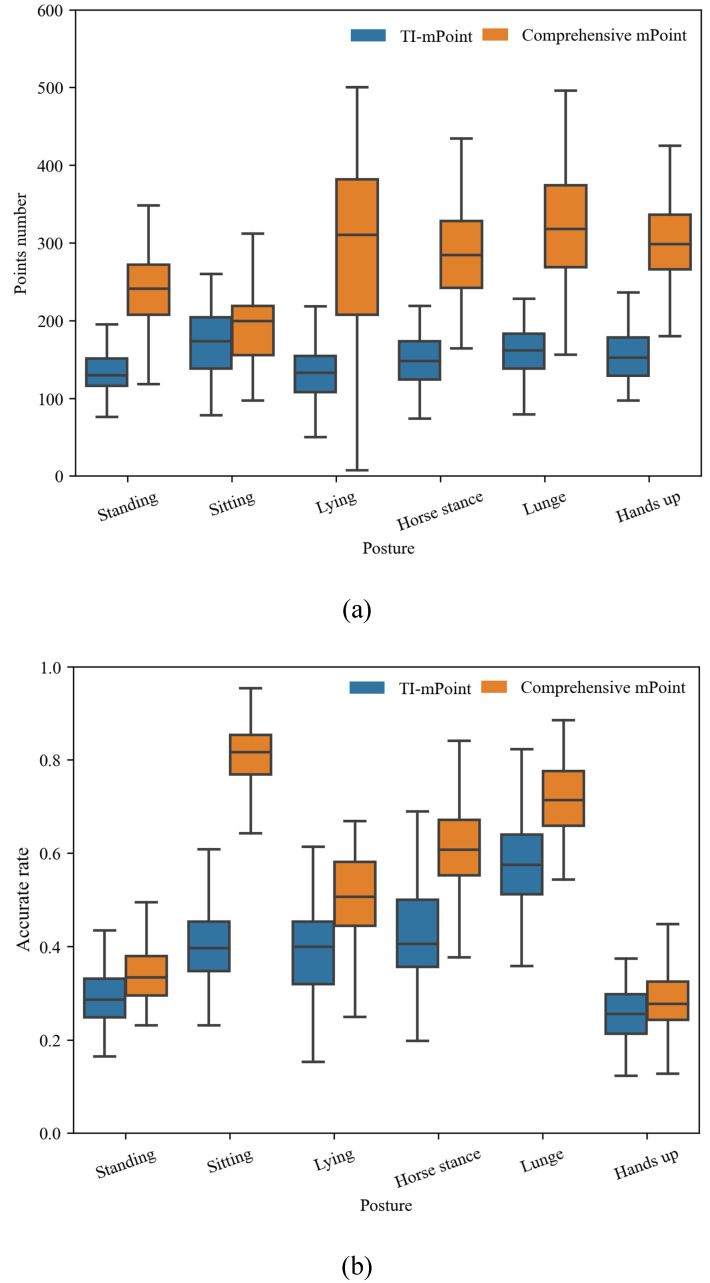
Box plot of the 100 sets of point cloud: (**a**) the relationship between points number and the posture and (**b**) the relationship between accuracy and the posture.

Moreover, 100 frames of original data for each posture were collected, and the point cloud data were calculated based on the TI-mPoint and comprehensive mPoint, respectively, for analyzation in this study. The points number and the accuracy rate of each set of point cloud are calculated according to the described Ac parameter. According to the results, the box plots of the points number and accuracy rate based on both methods are shown in Figure 13, where (a) shows the number of all detected points and (b) shows the accuracy rate of the point cloud results.

In box plot Figure 13, the height of the box is the Interquartile Range (IQR) value, which is positively correlated with the variability of the data. In the box plot of points number and accuracy rate, the median value of the comprehensive mPoint is obviously higher than TI-mPoint, indicating that the method proposed in this paper produces denser and more accurate point cloud. In the Sitting posture, due to the small reflection area of the human body, the points number of the comprehensive mPoint result has not increased substantially. However, the accuracy rate has almost doubled due to the large width of the posture cube. It also proved that the point cloud generated by the new method has a higher accuracy rate on the *Y*-axis. In the Hands up posture, due to the large reflection area of the human body, the points number of the comprehensive mPoint result almost doubled.

**Figure 14:**
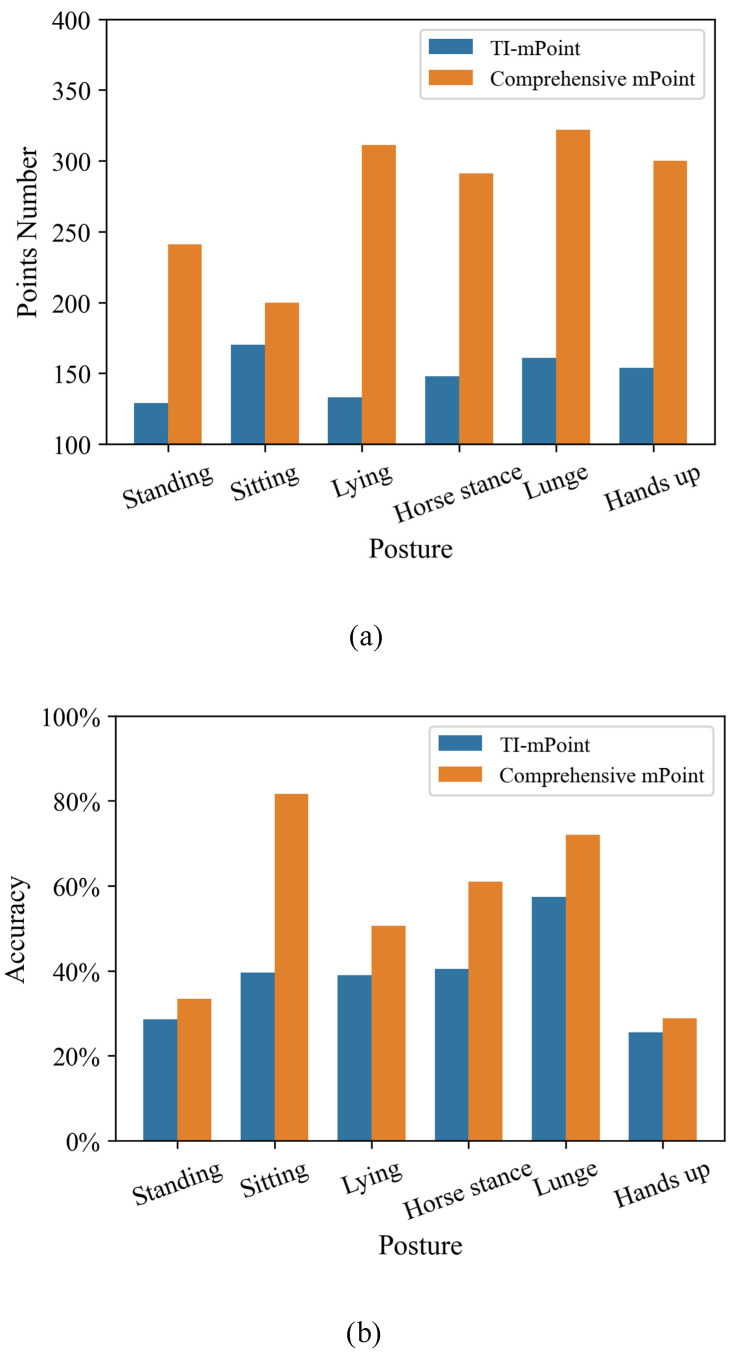
The histogram of the averaged value for 100 sets of point cloud: (**a**) the relationship between points number and the posture and (**b**) the relationship between accuracy rate and the posture.

In addition, for each posture, the averaged values of all the detected points number and accuracy rate of 100 sets of point cloud are calculated in order to better compare the results of the two different methods, as shown in Figure 14.

In terms of points number, the TI-mPoint achieved the least number of points during the Lying posture (133) and achieved the greatest number of points during the Sitting posture (170). The average number of points obtained on the six postures is 149. The comprehensive mPoint obtained the lowest number of points for the Sitting posture (200), the most points for the Lunge posture (322) and the average number of points obtained for the six postures is 278. The averaged value of comprehensive mPoint points number in the six postures is 86% higher than that of the TI-mPoint. In terms of accuratcy rate, TI-mPoint achieved a minimum value of 25.52% for the Hands up posture and a maximum value of 57.4% for the Lunge posture. The average value obtained on the six postures is 38.4%. The comprehensive mPoint achieved a minimum of 28.87% for the Hands up posture, a maximum of 81.6% for the Sitting posture and an average of 54.6% for the six postures. The accuracy rate of comprehensive mPoint for the average of the six postures is 42% higher than the TI-mPoint. The main reason for this is that the comprehensive mPoint not only considers the movement part of the human target but also the reflection information of the human body surface. Therefore, when a larger target reflection area is considered, a denser point cloud is obtained. This is consistent with the distribution of the points number for the six postures. In terms of accuracy rate, the results indicate that the longer the thickness of human posture cube on the *Y* axis, the higher the accuracy rate, illustrating that the precision of the point cloud on the *Y* axis needs to be improved. Furthermore, the running results show that the average running time of the TI-mPoint is 2.254 s, and the running time of the comprehensive mPoint is 2.291 s. The processing time has only increased by 1.6% as a slight tradeoff.

## 4. Conclusions

In this paper, an efficient 3D point cloud generation method for human targets with FMCW MIMO mm-wave radar was proposed. Compared with the commonly known TI-mPoint method, the proposed method creatively integrates various information of radar data, including Range-FFT, RDI, MTI results, RAI, etc., and realizes the point cloud generation system of the human bodies. By simultaneously benefiting from the considered human body movement in optimized RAI and human body reflection power in direct RAI, comprehensive human body surface information can be obtained in combined RAI for generating high density and precision point clouds. The method was explained in more detail on two human target data sets.

At the same time, the test was conducted with respect to six different postures of the tester. Compared with the TI-mPoint, the points number and accuracy rate of the point cloud generated by comprehensive mPoint increased by 86% and 42%, respectively. Thus, the density and accuracy rate of the point cloud greatly increased. Moreover, the proposed method also reduced the influence of multipath effects and did not substantially increase computational costs.

It is believed that the mm-wave radar, as a kind of non-intrusive technology, has excellent performance in penetrating common material in the room, and it is robust against non-line-of-sight interference and does not cause privacy issues. In the future, the application of point clouds generated by comprehensive mPoint could be used for practical products such as people counting, human identification recognition and posture classification.

## Figures and Tables

**Table 1:** Radar parameters.

Parameter	Configuration
Start frequency	60 GHz
Sweep bandwidth	3.92 GHz
Sweep slope	98 MHz/μs
Frame rate	5 fps
Sampling frequency	2200 ksps
Number of samples per chirp	64
Number of chirps per frame	200

**Table 2:** Posture cube with six different postures.

Human Posture	Dimension		
	Height/m	Width/m	Thickness/m
Standing	1.75	0.5	0.25
Sitting	1.28	0.55	0.65
Lying	0.7	1.45	0.48
Horse stance	1.6	0.6	0.4
Lunge	1.6	1.23	0.35
Hands up	1.71	1.75	0.25

## Data Availability

The data presented in this study are available upon request from the corresponding author.

## Nomenclature

FMCW	Frequency-Modulated Continuous Wave
MIMO	Multi-Input Multi-Output
Comprehensive mPoint	The point cloud generation method of millimeter wave radar proposed in this paper
TI-mPoint	The point cloud generation method of millimeter wave radar proposed in https://training.ti.com. Accessed 25 August 2021.
PIR	Passive Infrared
SAR	Synthetic Aperture Radar
FFT	Fast Fourier Transform
RCS	Radar Cross Section
Range-FFT	The FFT performed on the radar signal in the time domain data
MTI	Moving Target Indication
MVDR	Minimum Variance Distortionless Response
RAI	Range Angle Image
CFAR	Constant False Alarm Rate
RDI	Range Doppler Image
TDM	Time-Division Multiplexing
AOA	Angle of Arrival
SNR	Signal to Noise Ratio
DAQ	Data Acquisition Systems
FPGA	Field Programmable Gate Array
Doppler-FFT	The FFT performed on the chirps’ data
DBSCAN	Density-based Spatial Clustering of Applications with Noise
IQR	Interquartile Range
